# Alterations of Static and Dynamic Functional Connectivity of the Nucleus Accumbens in Patients With Major Depressive Disorder

**DOI:** 10.3389/fpsyt.2022.877417

**Published:** 2022-05-09

**Authors:** Bingqian Zhou, Yuan Chen, Ruiping Zheng, Yu Jiang, Shuying Li, Yarui Wei, MengZhe Zhang, XinYu Gao, Baohong Wen, Shaoqiang Han, Jingliang Cheng

**Affiliations:** ^1^Department of Magnetic Resonance Imaging, The First Affiliated Hospital of Zhengzhou University, Zhengzhou, China; ^2^Department of Psychiatry, The First Affiliated Hospital of Zhengzhou University, Zhengzhou, China

**Keywords:** major depressive disorder, resting-state functional connectivity, dynamic functional connectivity, nucleus accumbens, reward

## Abstract

**Background:**

Major depressive disorder (MDD) is associated with dysfunction of the reward system. As an important node in the reward system, the resting-state functional connectivity of the nucleus accumbens (NAc) is related to the etiology of MDD. However, an increasing number of recent studies propose that brain activity is dynamic over time, no study to date has examined whether the NAc dynamic functional connectivity (DFC) is changed in patients with MDD. Moreover, few studies have examined the impact of the clinical characteristics of patients with MDD.

**Methods:**

A total of 220 MDD patients and 159 healthy controls (HCs), group-matched for age, sex, and education level, underwent resting-state functional magnetic resonance imagining (rs-fMRI) scans. Seed-based resting-state functional connectivity (RSFC) and DFC of the NAc were conducted. Two sample *t*-tests were performed to alter RSFC/DFC of NAc. In addition, we examined the association between altered RSFC/DFC and depressive severity using Pearson correlation. Finally, we divided patients with MDD into different subgroups according to clinical characteristics and tested whether there were differences between the subgroups.

**Results:**

Compared with the HCs, MDD patients show reduced the NAc-based RSFC with the dorsolateral prefrontal cortex (DLPFC), hippocampus, middle temporal gyrus (MTG), inferior temporal gyrus (ITG), precuneus, and insula, and patients with MDD show reduced the NAc-based DFC with the DLPFC, ventromedial prefrontal cortex (VMPFC), ventrolateral prefrontal cortex (VLPFC), MTG, ITG, and insula. MDD severity was associated with RSFC between the NAc and precentral gyrus (*r* = 0.288, *p* = 0.002, uncorrected) and insula (*r* = 0.272, *p* = 0.003, uncorrected).

**Conclusion:**

This study demonstrates abnormal RSFC and DFC between the NAc and distributed cerebral regions in MDD patients, characterized by decreased RSFC and DFC of the NAc connecting with the reward, executive, default-mode, and salience network. Our results expand previous descriptions of the NAc RSFC abnormalities in MDD, and the altered RSFC/DFC may reflect the disrupted function of the NAc.

## Introduction

Major depressive disorder (MDD) is the second leading cause of disability worldwide now, and it will be the most common and economically burdening disease in the world by 2030 (1). Although intensive neurobiological research has been carried out for nearly 60 years, our understanding of MDD’s pathophysiology remains limited (2, 3). The study of functional connectivity is based on resting-state functional magnetic resonance imaging (rs-fMRI) as an established technique for unbiased analysis of the brain’s functional connectome (4, 5). The dysfunction of the brain’s connectome may be related to the symptoms of MDD. At present, a large number of studies using rs-fMRI functional connectivity have been carried out, which is expected to become an objective neurobiological marker.

The past neuroimaging studies summarized the unique functional systems of the brain regions. Huckins et al. (6) used the resting-state functional connectivity (RSFC) method based on seed and graph theory to describe the system-level organization of static putative reward regions, and they found the regions related to rewards formed a system of priority coupling at rest. Additionally, a recent meta-analysis by Bartra et al. (7) confirmed that the core reward network including the anterior cingulate cortex (ACC), the orbital prefrontal cortex (OFC), the ventral striatum (VS), the ventral tegmental area (VTA), the amygdala, and other brain regions. The independent behavioral studies and neuroimaging studies (including those using various task paradigms) over the past decade have accumulated a great deal of evidence that MDD is associated with dull reward signals in the reward system (8–11). In patients with MDD, abnormal reward processing is primarily thought to be associated with the disruption of mesolimbic striatum circuitry (12). For example, decreased functional connectivity has been manifested among the VTA, striatum, and VMPFC (13). Notably, the nucleus accumbens (NAc) is not only an important node of the reward network (14, 15) but also an important brain substructure of neuroregulation, especially in the reward and addictive behavior loop. The NAc mainly mediates the hedonic perception of rewards and is concerned with reward assessment and expectation (16). Besides, the NAc is involved in complex interactions between the dopamine, serotonin, and glutamatergic systems, and it also has complex functional connectivity with the limbic system and prefrontal cortex (17–21). In recent years, there has been an increase in studies on the role of the NAc in emotion and cognition regulation, especially in affective disorders, such as MDD (21–23).

Converging evidence suggests that the functional connectivity of NAc with other brain regions could be a potentially valuable biomarker for MDD (12, 21, 22, 24, 25), but not all the experimental results are consistent. As noted, there have been reports of both increased and decreased striatum-PFC functional connectivity in patients with MDD compared to HCs. For example, Gabbay et al. (12) found increased striatal-PFC functional connectivity, but Furman et al. (26) reported decreased striatum-PFC functional connectivity for ventral striatum seeds. The inconsistency may result from the limited statistical power of small samples. Inconsistencies in the literature have prompted researchers to conduct more in-depth analyses to identify common abnormal activation patterns involving the NAc during reward processing. Most of the previous studies on MDD reward system used the method of functional connectivity in the resting state (27). However, based on the assumption that the brain signal is relatively static during the whole data scanning period, this method ignores the dynamics of the brain and the time-varying functional connections between brain regions, that is, this method ignores that functional connection changes over time (28).

Therefore, the recently emerged analytic method, sliding window correlation analysis, has been used to reveal the dynamic of functional connectivity (DFC) by measuring the temporal variability of functional connectivity. Recently, increasing research has focused on DFC, and some researchers have found profound indicators related to DFC (29, 30). Dynamic characteristics of brain activity are sensitive to human behavior and brain development (31–33). In addition, DFC has been widely used to explore the pathological mechanisms of other mental diseases, such as schizophrenia, epilepsy, autism, and anxiety, and studies have shown that DFC can provide reliable potential biomarkers for the diagnosis of mental diseases (34–37). Despite the numerous RSFC studies in recent years, there lacks research on the DFC of the NAc in the reward network of patients with MDD. By combining the RSFC and DFC model of the NAc in patients with MDD, we may be able to promote the understanding of the pathogenesis of MDD from the perspective of time variability.

## Materials and Methods

### Participants

A total of 220 patients with MDD and 159 matched healthy controls (HCs) were enrolled at the First Affiliated Hospital of Zhengzhou University. The current study was authorized by the ethics committee of the first affiliated Hospital of Zhengzhou University. Written informed consents were gotten from all participants.

According to the Structured Clinical Interview of DSM-IV(SCID), which was independently evaluated by two qualified psychiatrists, the patient was clinically diagnosed with severe depressive disorder. All patients with MDD met the following criteria: (1) two professional psychiatrists use DSM-IV to diagnose bipolar or unipolar depressive disorder; (2) right-handed; (3) age in the range of 18–55 years; The patients with MDD were excluded, if they (1) are suffering from other mental illness; (2) are suffering from diseases other than mental or neurological diseases, such as cardiovascular disease; (3) have major physical diseases; (4) do drug abuse or drug addiction; and (5) have brain damage. In addition, exclusion criteria for healthy controls include the following: (1) family history of hereditary neurological disorders; (2) loss of consciousness resulting from head injury; (3) alcohol or substance abuse; and (4) any metallic objects in their body.

### Data Acquisition

All magnetic resonance imaging (MRI) data were obtained using 3.0TMR scanners (GE discovery 750, America) and standard head coils. Before scanning, a foam-filled head coil is used to reduce the movement of the subject’s head. To reduce the impact of machine noise on the subjects during the scanning process, we provided earplugs to the subjects to isolate the noise to the maximum extent. Before the scanning, the subjects were told to keep their head still and close their eyes without thinking about anything. The imaging parameters were as follows: repetition time (TR)/echo time (TE) = 2,000/40 ms; flip angle = 90 degrees; field of view (FOV) = 22 × 22 cm^2^; matrix = 64 × 64; slices = 32; slice thickness = 3.0 mm; gap = 0.5 mm.

### Data Preprocessing

We used the Data Processing and Analysis for (Resting-State) Brain Imaging(DPABI v4.3)^1^ to process Neuroimaging data. In order to stabilize the MR signal, the first five volumes of each participant are discarded. Resting-state images were first subjected to slice time correction and realignment. Then, we matched the functional image to the standard template (Montreal Neurological Institute) and the standardized image voxel is 3 mm × 3 mm × 3 mm. The subjects whose head motion parameters are greater than 3.0 mm and 3.0 degrees are excluded. Then, the data were smoothed with a Gaussian kernel (6 mm full width at half maximum), and the image was detrended to reduce low-frequency drift. In order to remove the low-frequency drift and high-frequency physiological noise, we choose the frequency range of the temporal band-pass filter to be 0.01∼0.10 Hz. We regressed Friston 24 motor parameters (38), white matter signal, and cerebrospinal fluid signal as interference covariates. However, in the current study, we did not regress the global signal, because the variance of the global signal of psychiatric disorders, including depression, and its topology have changed (39). After despiking, the outliners were replaced with the best estimate using a third-order spline fit to clean up the time course portions. Outliners were detected based on the median absolute deviation, as implemented in 3dDespike^2^ (40). We also calculated the mean frame displacement (FD).

### Seed Based and Dynamic and Static Functional Connectivity Analysis

A previously validated bilateral NAc (X = ±9, Y = 9, Z = –8) was selected as the seed (41), the seed is defined as a 4 mm diameter sphere surrounding a central voxel. REST software was used to calculate the seed-based RSFC. The spatially averaged mean value of each seed was extracted. A Pearson correlation analysis was conducted between the mean time series of the seed region and the time series of each voxel of the whole brain. Finally, in order to improve the normality, correlation coefficients were transformed to Z-map with Fisher’s r-to-z transformation.

The DFC was computed using a sliding window approach *via* DynamicBC (v1.1)^3^ (42). The DFC divides all the time points of the subjects into different time windows according to a certain length and then calculates the correlation between time series based on a series of time windows. One of the resting-state DFC important parameters is the window length. According to the previous literature (43), the length of the sliding window was set at 50 TRs, and the windows overlap was set at 90%. Then, seed-based DFC analysis was performed for each window, that is, Pearson correlation coefficients between the time series of the bilateral NAc and whole brain voxels were calculated, and a correlation map of each window was established. Fisher’s r-to-z transformation was applied to convert the correlation coefficient map into the z-map to improve the normality. For each subject, we calculated the variance of functional connectivity in each time window and finally obtained the functional connectivity variance map.

### Impact of Clinical Characteristics on Functional Connectivity

Because of the clinical characteristics diversity of our data, in addition to comparing all the MDD patients with healthy controls, we also subdivided the patients with MDD into different subgroups. The details are as follows: female and male; adolescent and adult; first-episode MDD and HCs; MDD with long-illness duration, medium duration, and with short illness duration.

### Statistical Analysis

The two-tailed two-sample *t*-test was used to determine altered RSFC and DFC of the NAc in patients with MDD compared with HCs. Age, gender, and years of education were used as covariates for regression. All results reported were thresholded using the Gaussian random field (GRF) correction with a voxel-level threshold of *p* < 0.001 and a cluster-level threshold of *p* < 0.05.

### Relation With Depressive Severity

To investigate the association between altered functional connectivity and depressive severity, we extracted the altered RSFC and DFC of peak coordinates with a spherical radius of 3 mm. In MDD patients, the spatial mean values of altered RSFC and DFC were then correlated with HAMD scores with Pearson correlation.

## Results

### Demographic and Clinical Information

As shown in Table 1, there was no statistical significance in age, gender, and education level between the MDD group and HC group (*p* > 0.05). Patients with MDD had higher scores on HAMD than those in the HC group.

**TABLE 1:** Characteristics of patients with MDD and healthy controls.

	MDD(*n* = 220)	HC(*n* = 159)	*p*
Age (years), Mean ± SD	22.66 ± 8.75	23.74 ± 8,67	0.11*^a^*
Gender, male: female	100:120	74:85	0.83*^b^*
Years of education, Mean ± SD	11.60 ± 3.00	13.00 ± 4.60	0.15*^a^*
Mean FD (mm)	0.11 ± 0.09	0.12 ± 0.09	0.23*^a^*
HAMD-24	36.00 ± 12.23	——	

*p^a^-value was obtained by a two-tailed two-sample t-test.*

*p^b^-value was obtained by χ^2^ two-tailed test.*

### Functional Connectivity Analysis

#### The Resting-State Functional Connectivity Analyses of the Nucleus Accumbens

**FIGURE 1:**
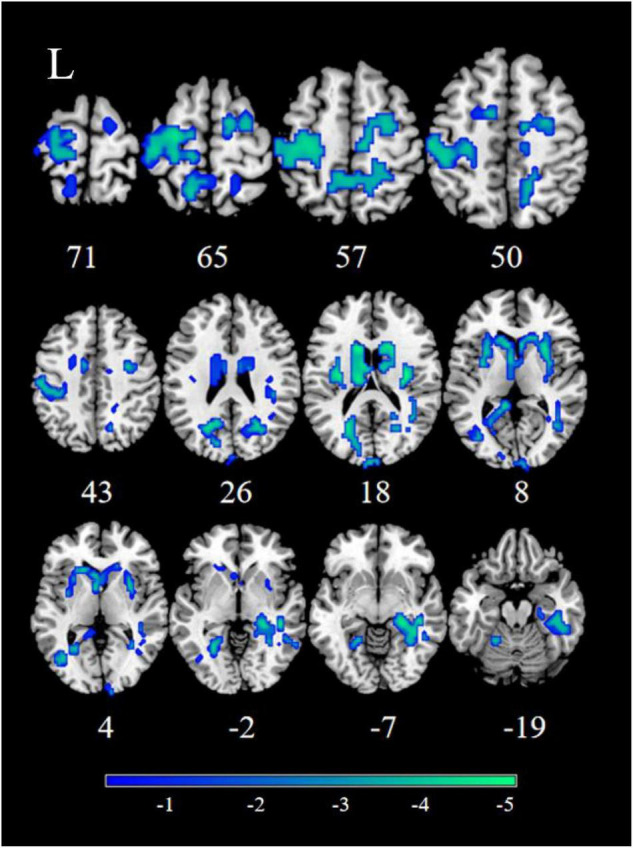
Significant decreased resting-state functional connectivity (RSFC) of the right nucleus accumbens (NAc) in patients, compared with healthy controls (HCs). The blue regions in the brain slices present the location of difference (GRF corrected).

**FIGURE 2:**
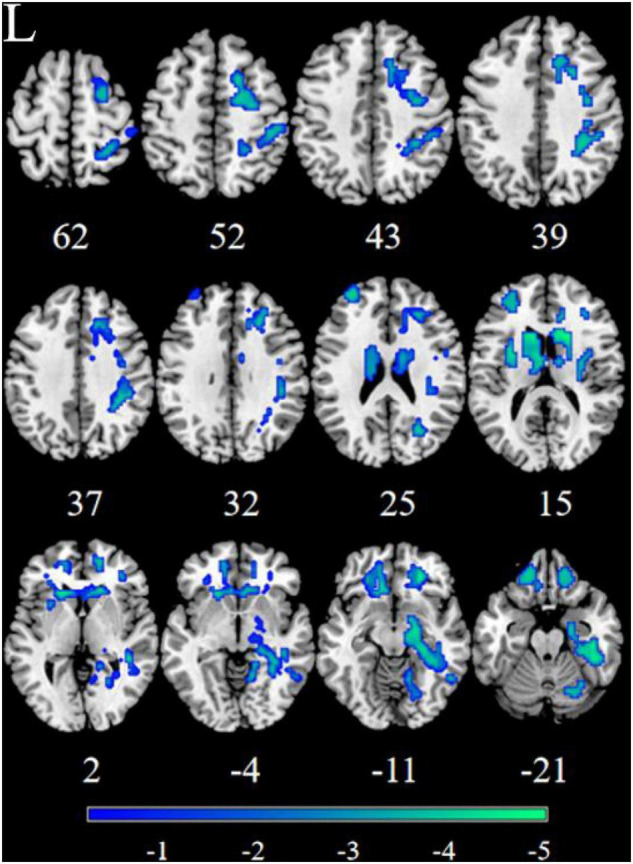
Significant decreased resting-state functional connectivity (RSFC) of the left nucleus accumbens (NAc) in patients, compared with HC. The blue regions in the brain slices present the location of difference (GRF corrected).

Patients with MDD exhibited decreased RSFC between the NAc and the Parahippocampal Gyrus (PHG), Hippocampus (HIP), Caudate (CAU), Thalamus (THA), Fusiform Gyrus (FFG), Insula (INS), Precuneus (PCUN), Inferior Frontal Gyrus (IFG), Lingual Gyrus (Ling), Superior Frontal Gyrus Orbital part (ORBsup), Middle Frontal Gyrus (MFG), Rectal Gyrus (REC), Superior Temporal Gyrus (STG), Middle Temporal Gyrus (MTG), Inferior Temporal Gyrus (ITG), Postcentral Gyrus (PoCG), and Precentral Gyrus (PreCG) (Figures 1, 2 and Table 2).

**TABLE 2:** Resting-State Functional Connectivity (RSFC) Group Comparisons (MDD vs. HCs).

			Cluster	Peak				
Seed	Connectivity peak	Hemisphere	Size	X	Y	Z	T	Cohen’s d
Right NAc	CAU	L	158	–12	12	18	–5	–0.52
	CAU	R	78	21	15	18	–4.55	–0.47
	INS	L	62	–33	–12	21	–5.17	–0.53
	INS	R	58	30	–18	18	–4.92	–0.51
	FFG	R	123	42	–36	–24	–4.1	–0.42
	PHG	R	56	27	–30	–12	–4.33	–0.45
	THA	L	46	–12	–15	18	–4.79	–0.49
	MTG	R	55	42	–51	12	–4.09	–0.42
	ITG	R	48	51	–39	–18	–3.83	–0.39
	HIP	R	47	27	–33	–6	–4.76	–0.49
	PUT	R	45	30	12	9	–4.64	–0.48
	PCUN	R	55	15	–56	49	–3.74	–0.39
	PCUN	L	96	–3	–48	60	–4.12	–0.42
	SOG	L	66	–21	–63	24	–4.34	–0.45
	PoCG	L	252	–42	–24	60	–4.27	–0.44
	PreCG	L	234	–24	–15	66	–4.63	–0.48
	SFG	R	86	27	–3	63	–4.12	
Left NAc	FFG	R	237	42	–29	–21	–4.35	–0.45
	PHG	R	108	24	–3	36	–4.47	–0.46
	ITG	R	80	54	–57	–9	–3.51	–0.36
	HIP	R	76	21	–12	–15	–4.47	–0.46
	CAU	L	150	–15	–15	21	–4.98	–0.51
	CAU	R	97	21	21	12	–5.26	–0.54
	MFG	L	102	–33	54	27	–4.46	–0.46
	SFG	R	137	21	27	33	–4.6	–0.47
	ORBsup	R	71	24	39	–12	–4.61	–0.47
	ORBsup	L	44	–17	42	–15	–3.89	–0.4
	INS	L	55	–30	7	15	–4.58	–0.47
	REC	L	32	–12	39	–18	–3.87	–0.4
	PoCG	R	77	48	–30	54	–3.76	–0.39
	INS	R	21	36	–6	18	–4.02	–0.41
	ACC	R	22	–15	33	19	–4.28	–0.44

#### The Dynamic Functional Connectivity Analyses of the Nucleus Accumbens

**FIGURE 3:**
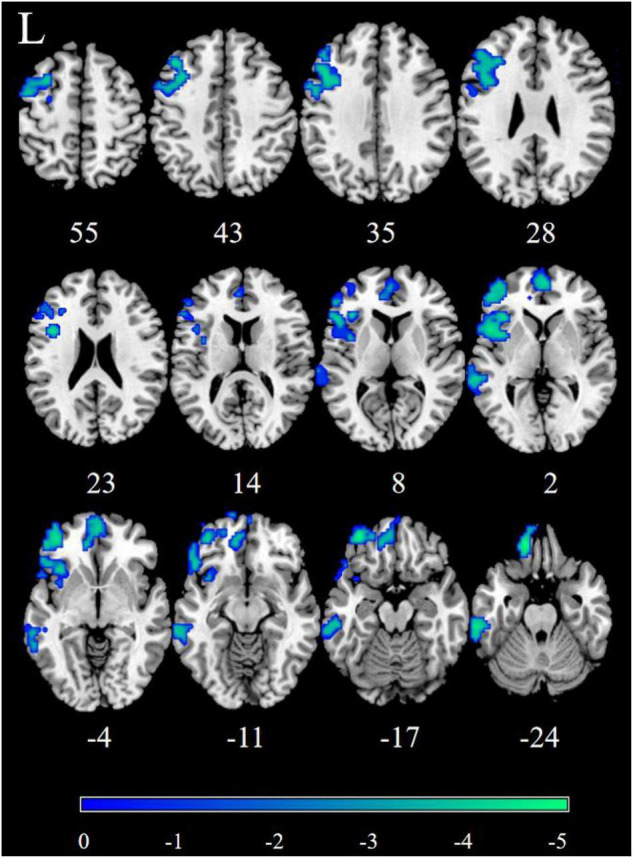
Significantly decreased dynamic functional connectivity (DFC) of the right nucleus accumbens (NAc) in patients, compared with HC. The blue regions in the brain slices present the location of difference (GRF corrected).

Patients with MDD exhibited decreased DFC between the NAc and the STG, MTG, ITG, FFG, MFG, Inferior Frontal Gyrus triangular part (IFGtriang), Inferior Frontal Gyrus orbital part (ORBinf), PreCG, Superior Frontal Gyrus (SFG), INS, Frontal Inferior Gyrus opercular part (IFGoperc), Superior Frontal Gyrus medial orbital (ORBsupmed), Superior Frontal Gyrus medial (SFGmed), REC, ORBsup, Anterior Cingulate Cortex (ACC), Inferior Parietal Lobule(IPL), Angular Gyrus (ANG), and PCUN (Figure 3 and Table 3).

**TABLE 3:** Dynamic Functional Connectivity (DFC) Group Comparisons (MDD vs. HCs).

			Cluster	Peak				
Seed	Connectivity peak	Hemisphere	Size	X	Y	Z	T	Cohen’s d
Right NAc	MTG	L	151	–63	–36	3	–4.34	–0.45
	ITG	L	95	–60	–30	–24	–4.58	–0.47
	MFG	L	302	–39	27	45	–4.44	–0.46
	IFGtriang	L	215	–48	42	0	–4.43	–0.46
	PreCG	L	130	–48	3	51	–4.11	–0.42
	ORBinf	L	109	–51	27	–9	–4.26	–0.44
	IFGoperc	L	85	–48	12	3	–4.80	–0.49
	INS	L	81	–39	6	3	–4.55	–0.47
	ORBsupmed	L	67	0	57	–3	–4.22	–0.43
	ORBsup	L	43	–15	42	–24	–5.21	–0.54
	REC	L	39	–12	45	–18	–3.93	–0.40
	SFGmed	L	36	0	57	1	–4.43	–0.46
	ACC	L	33	–12	42	–3	–4.09	–0.42
	SFGmed	R	21	3	54	3	–4.13	–0.43

*CAU, caudate; INS, insula; FFG, fusiform; THA, thalamus; MTG, middle temporal gyrus; ITG, inferior temporal gyrus; HIP, hippocampus; PUT, putamen; PCUN, precuneus; SOG, superior occipital gyrus; PoCG, postcentral gyrus; PreCG, precentral gyrus; SFG, superior frontal gyrus; MFG, middle frontal gyrus; PHG, parahippocampal; ORBsup, superior frontal gyrus, orbital part; REC, rectus; ACC, anterior cingulate cortex; IFGtriang, inferior frontal gyrus, triangular part; ORBinf, inferior frontal gyrus, orbital part; ORBsupmed, superior frontal gyrus, medial orbital; IF Goperc, inferior frontal gyrus, opercular part; SFGmed, superior frontal gyrus, medial.*

### The Effect of the Clinical Characteristics on the Dynamic and Static Functional Connectivity

We removed recurrent MDD, leaving only first-episode MDD patients and HCs, and eventually, we found that almost all of the brain areas in the all MDD data results were disappearing, such as MFG, ORBsupmed, ACC, HIP, SFGmed. Then, based on gender and age, we divided depressed patients into male patients with MDD and female patients with MDD, adult patients with MDD patients, and adolescent patients with MDD. Finally, according to illness duration, we classified patients with MDD as long duration (≥12 months), medium duration (3–12 months), and short duration (≤3 months), we did not find any significant differences between these groups.

### Decreased Resting-State Functional Connectivity of the Nucleus Accumbens Was Correlated With the HAMD Scores

**FIGURE 4:**
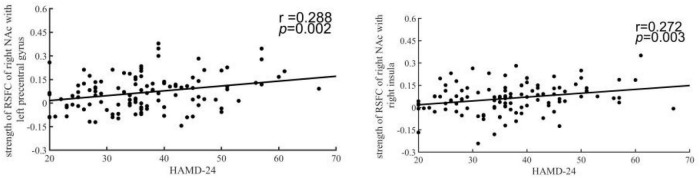
The HAMD-24 scores were correlated with decreased resting-state functional connectivity (RSFC) of the nucleus accumbens (NAc). The y-coordinate meant HAMD-24 scores. The x-coordinate meant the z-score of RSFC in patients.

To explore the relationship between the dysfunction of the NAc and patients’ symptoms, we examined the correlation between the altered RSFC and DFC strength and HAMD-24 scores. In the results, the strength of the left NAc-right insula RSFC (*r* = 0.272, *p* = 0.003 uncorrected) and the right NAc-right precentral gyrus was significantly correlated with HAMD scores (*r* = 0.288, *p* = 0.002 uncorrected) (Figure 4).

## Discussion

By conducting a comprehensive large sample analysis, we revealed specific patterns of abnormalities in the NAc-based network in patients with MDD, and to our knowledge, this study is the first to explore differences in DFC of the NAc to the whole brain between MDD patients and healthy controls, while analyzing the effects of different clinical features. We found that in MDD patients, the NAc-based RSFC and DFC abnormalities were mainly located within the OFC/VMPFC, VLPFC, DLPFC, precuneus, and some regions of the temporal lobe, such as STG, MTG, ITG, compared with HC. In addition, when we directly compared the two groups of patients with MDD (first episode MDD and all MDD), we found that the difference was mainly caused by the recurrent MDD patients, but it was not related to age, gender, and illness duration.

In this study, the reduced NAc-VMPFC/OFC RSFC and DFC were found in patients with MDD, which is both inconsistent and consistent with previous studies reporting changed NAc-VMPFC/OFC RSFC in patients with MDD (26). Using different methodological approaches may be the main reason for the difference in results. Clinical characteristics of MDD patients and sample size may also play a role in the differences. We found that the functional connectivity of the NAc with the OFC/VMPFC was decreased in the patients with MDD. The NAc, VMPFC, and OFC are important hubs for reward circuits (44). Human fMRI studies have shown that activity between VMPFC/OFC and VS is highly correlated in resting states or in tasks involving rewards (45–47). The VMPFC and OFC are two overlapping subregions of PFC, which constitute the inferior medial wall and ventral side of the frontal lobe, respectively (44). Some neurological cases report damage to the VMPFC/OFC leading to significant impairment in dealing with risk, uncertainty, reward, and punishment (48). At present, a series of paradigm studies, including risk gambling and probabilistic reinforcement learning, have recorded the value-based decision-making defects of patients with the VMPFC disease (49–51). A study reported less activation of VMPFC in depressed patients under negative conditions (52). Animal studies have also proved the important role of the OFC in coding reward values (53–55). In addition, Cheng et al. (56) showed that functional connectivity between the lateral OFC and precuneus was decreased, these findings confirmed a theory that the non-reward system in the lateral OFC is associated with the poor self-esteem in depression. Decreased functional connectivity between the NAc and VMPFC/OFC may reflect low reward function in patients with MDD. In the mid-1960s, it was proposed that depression was associated with decreased levels of the central nervous system neurotransmitter, dopamine (57). The NAc, VMPFC, and OFC are all important projection areas for dopamine (58). This reduction in functional connectivity strength may be associated with a decrease in dopamine levels. Rodent studies have demonstrated that there is a direct glutamatergic projection from VMPFC/OFC to VS (59, 60), MDD patients had significantly higher glutamate levels compared to HCs and resulted in neuronal atrophy (61). Decreased strength of the NAc and VMPFC/OFC functional connectivity may also be associated with increased glutamate levels.

In addition, the VMPFC is a key node of the Default mode network (DMN) (62), which is consistent with previous studies that the reward defect is associated with decreased connectivity between the NAc and the DMN (21, 63). In addition to the VMPFC, decreased RSFC of the NAc with the precuneus and hippocampus, which are also key nodes of the DMN (62), was also found in this study. The DMN is involved in spontaneous cognition, self-referential processing, and affective decision making (64). For instance, the precuneus is involved in cognitive control functions including visual imagery and self-orientation processes and is further engaged in the integration of mental processing (65). Multiple investigations have repeatedly demonstrated the abnormality of DMN in patients with MDD (66–68). Indeed, a previous investigation of the striatum demonstrated a negative correlation between the strength of the caudate/NAc-precuneus/PCC FC and depression severity (12), and they concluded that the caudate was associated with cognition and reward (45). Decreased RSFC between the NAc and the DMN may reflect impairments in reward-oriented internal cognition, which may be a phenotype of depression.

We also observed significantly reduced RSFC and DFC between the DLPFC and the NAc in patients with MDD compared to HC. The DLPFC is a key node in the central executive network (69), and the DLPFC is primarily involved in emotion regulation and cognitive function (70), which play an important role in the pathophysiology of depression. In addition, the DLPFC is involved in the volitional regulation of emotions, and it is critical in the reassessment of emotional stimuli in healthy children, adolescents, and adults (71, 72). As noted, there have been reports of decreased NAc-DLPFC RSFC in patients with MDD compared to HC (21). Another study found that in response to reward cues following positive emotion induction, higher anhedonia among patients with MDD was associated with decreased RSFC between the DLPFC and the right NAc (15), anhedonia is a core symptom of MDD and has been described as an endophenotypic marker of MDD (73). Two previous rs-fMRI studies reported that depressive adults displayed reduced ReHo in the DLPFC (74, 75). The DLPFC is involved in the reassessment of emotional stimuli and the regulation of emotions, together with the observed reduction in the NAc-DLPFC RSFC and DFC, suggests that depressed patients have a disrupted network of brain areas responsible for emotion regulation and are unable to recognize the positive components of emotional stimuli, which may contribute to the etiology of depression.

In addition, we found decreased RSFC of the NAc with the insula and decreased DFC of the NAc with the VLPFC. The VLPFC is associated with reward processing and it is abnormally activated in patients with Bipolar Disorder (76–78). The insula is the hub of large-scale brain networks interaction that is involved in externally oriented attention and self-cognition (65), one study found that the insula is associated with the value of action, and the insula is associated with seeking reward-related decisions (79). Thus, the abnormalities in the functional connectivity of the NAc with the VLPFC and insula may be that they do not support NAc function. The study confirmed increased activation of the VLPFC and insula in response to social punishment in depressed patients (80–82). The striatum, VLPFC, and insula also show strong activation in tasks involving monetary and social stimulation (83, 84). Abnormalities in the VLPFC and insula have been shown to be responsible for depressive information processing deficits due to impaired emotion salience processing and adverse emotion regulation (84). The NAc, VLPFC, and insula all belong to salience network (SN), and Conio et al. (85) proposed a model in which patients with MDD patients have lower dopamine levels that lead to decreased RSFC within the SN, which is also consistent with our results. Correspondingly, our results showed that RSFC strength between the left NAc and the insula is positively correlated with depression severity. Indeed, increased RSFC between the NAc and insula is associated with lower reward sensitivity in a variety of psychiatric disorders (86), therefore, it may represent a transdiagnostic marker of reward dysfunction.

Similarly, the RSFC of the NAc with the supplementary motor area (SMA) was decreased, which is a key node in the reward network. In reward task-based fMRI studies, it has been found that the SMA is activated during the anticipation/decision-making phase of the reward (87). We also found positive correlations between the NAc-precentral gyrus RSFC strength and depression severity; this finding is akin to a previously reported correlation between striatum-SMA RSFC strength and anhedonia severity in adolescents with MDD (12). Overall, these findings suggest that the NAc-SMA connectivity is disturbed in patients with MDD. We likewise found reduced DFC of the NAc with MTG and ITG, and this is consistent with previous studies. For example, Yang et al. (88) found that lower RSFC between caudate and MTG was associated with more severe anhedonia. Similarly, Gabbay et al. (12) found decreased RSFC between NAc and MTG.

This is the first study to examine alterations of the NAc with whole brain functional connectivity in patients with MDD by combining static and dynamic methods. Compared with separate static or dynamic functional connectivity, our results show that the combination of RSFC and DFC can provide more effective information, this is consistent with previous studies that RSFC and DFC provide complementary information (89–91), and they may reflect different brain dynamics, and both can be used to explore the pathophysiological mechanisms of depression. These differences in results may be due to the fact that RSFC is an average analysis of the whole time series and cannot capture the instantaneous change in the strength of connections between brain regions, but the DFC analysis calculates the connectivity of the overlapping time periods in the whole time series and then determines the variance of the correlation. In addition, a combination of dynamic and static functional connectivity has been used in schizophrenia and bipolar disorder (92).

On the basis of all MDD data, we further grouped patients according to clinical characteristics. We removed recurrent MDD, leaving only first-episode MDD and HCs, and eventually, we found that almost all of the brain areas in the all MDD data results were disappearing. This may indicate that recurrent MDD is the main cause of altered brain FC. One study demonstrated decreased RSFC of the DMN in recurrent MDD (93). Similarly, another study found that hippocampal volume reduction (a key DMN node) was only found in recurrent MDD, but not in first-episode MDD (94). This may be due to the different illness duration and medication statuses of first-episode MDD vs. recurrent MDD. Since we did not have information on the illness duration of recurrent MDD, we divided the first-episode MDD into three groups based on the illness duration: long (≥12 months), medium (3–12 months), and short(≤3 months). However, in the direct comparison, illness duration was not related to RSFC and DFC.

There are still some potential limitations of this study. First, a small proportion of patients with MDD in this study were taking medications and antidepressant drugs may affect brain functional connectivity in these patients. Therefore, future studies should be conducted on the first episode, drug naïve patients with MDD. Second, we lack information on the illness duration of recurrent MDD, we should examine in more detail the effect of illness duration on brain function in recurrent MDD. Third, the sample size of recurrent patients with MDD is small, in the future, it will be necessary to include more recurrent patients to determine the relationship between group differences and the number of depressive episodes; Fourth, this study is cross-sectional research. Therefore, longitudinal studies are needed to determine whether the dynamic and static functional connectivity of the NAc to the whole brain is altered during depressive episodes. Finally, we did not measure participants’ levels of drowsiness when scanning at rest, this has been shown to affect DFC.

In conclusion, this study demonstrates abnormal RSFC and DFC between the NAc and distributed cerebral regions in patients with MDD, characterized by decreased RSFC and DFC of the NAc connecting with the reward, executive, default-mode, salience network, and temporal lobe. Our findings extend previous literature and provide further evidence for a critical role of the NAc in the neuropathology of depression, and potential targets for alternative interventions or monitoring treatment response are indicated.

## Data Availability Statement

The datasets presented in this article are not readily available because, the dataset is for internal use only by the MRI Department of the First Affiliated Hospital of Zhengzhou University. Requests to access the datasets should be directed to JC.

## Ethics Statement

The studies involving human participants were reviewed and approved by Scientific Research and Clinical Trial Ethics Committee of the First Affiliated Hospital of Zhengzhou University. Written informed consent to participate in this study was provided by the participants’ legal guardian/next of kin. Written informed consent was obtained from the individual(s), and minor(s)’ legal guardian/next of kin, for the publication of any potentially identifiable images or data included in this article.

## Author Contributions

BZ conceived and designed the study, analyzed the data, and took responsibility for the manuscript. BZ, YC, and SH supervised the conduct of the study and drafted the initial manuscript. BZ, YC, YJ, and SL were responsible for data acquisition. BZ, RZ, YC, and YJ assisted with the literature review. JC and SH reviewed and revised the manuscript. All authors read and approved the final manuscript.

## Conflict of Interest

The authors declare that the research was conducted in the absence of any commercial or financial relationships that could be construed as a potential conflict of interest.

## Publisher’s Note

All claims expressed in this article are solely those of the authors and do not necessarily represent those of their affiliated organizations, or those of the publisher, the editors and the reviewers. Any product that may be evaluated in this article, or claim that may be made by its manufacturer, is not guaranteed or endorsed by the publisher.

## Footnotes

1
http://rfmri.org/DPABI

2
http://afni.nimh.nih.gov/afni

3
www.restfmri.net/forum/DynamicBC

## References

[1] Ferrari AJ Charlson FJ Norman RE Patten SB Freedman G Murray CJ Burden of depressive disorders by country, sex, age, and year: findings from the global burden of disease study 2010. *PLoS Med.* (2013) 10:e1001547. 10.1371/journal.pmed.1001547 24223526 PMC3818162

[2] Arroll B Elley CR Fishman T Goodyear-Smith FA Kenealy T Blashki G Antidepressants versus placebo for depression in primary care. *Cochrane Database Syst Rev.* (2009) 8:Cd007954. 10.1002/14651858.CD007954 19588448 PMC10576545

[3] Mojtabai R. Clinician-identified depression in community settings: concordance with structured-interview diagnoses. *Psychother Psychosom.* (2013) 82:161–9. 10.1159/000345968 23548817

[4] Gorges M Müller HP Lulé D Ludolph AC Pinkhardt EH Kassubek J. Functional connectivity within the default mode network is associated with saccadic accuracy in Parkinson’s disease: a resting-state FMRI and videooculographic study. *Brain Connect.* (2013) 3:265–72. 10.1089/brain.2013.0146 23627641

[5] Lang EW Tomé AM Keck IR Górriz-Sáez JM Puntonet CG. Brain connectivity analysis: a short survey. *Comput Intell Neurosci.* (2012) 2012:412512. 10.1155/2012/412512 23097663 PMC3477528

[6] Huckins JF Adeyemo B Miezin FM Power JD Gordon EM Laumann TO Reward-related regions form a preferentially coupled system at rest. *Hum Brain Mapp.* (2019) 40:361–76. 10.1002/hbm.24377 30251766 PMC6865378

[7] Bartra O McGuire JT Kable JW. The valuation system: a coordinate-based meta-analysis of BOLD fMRI experiments examining neural correlates of subjective value. *Neuroimage.* (2013) 76:412–27. 10.1016/j.neuroimage.2013.02.063 23507394 PMC3756836

[8] Forbes EE Shaw DS Dahl RE. Alterations in reward-related decision making in boys with recent and future depression. *Biol Psychiatry.* (2007) 61:633–9. 10.1016/j.biopsych.2006.05.026 16920074

[9] Steele JD Kumar P Ebmeier KP. Blunted response to feedback information in depressive illness. *Brain.* (2007) 130:2367–74. 10.1093/brain/awm150 17586866

[10] Kumar P Waiter G Ahearn T Milders M Reid I Steele JD. Abnormal temporal difference reward-learning signals in major depression. *Brain.* (2008) 131:2084–93. 10.1093/brain/awn136 18579575

[11] Kumar P Goer F Murray L Dillon DG Beltzer ML Cohen AL Impaired reward prediction error encoding and striatal-midbrain connectivity in depression. *Neuropsychopharmacology.* (2018) 43:1581–8. 10.1038/s41386-018-0032-x 29540863 PMC5983542

[12] Gabbay V Ely BA Li Q Bangaru SD Panzer AM Alonso CM Striatum-based circuitry of adolescent depression and anhedonia. *J Am Acad Child Adolesc Psychiatry.* (2013) 52:628–41.e13. 10.1016/j.jaac.2013.04.003 23702452 PMC3762469

[13] Downar J Geraci J Salomons TV Dunlop K Wheeler S McAndrews MP Anhedonia and reward-circuit connectivity distinguish nonresponders from responders to dorsomedial prefrontal repetitive transcranial magnetic stimulation in major depression. *Biol Psychiatry.* (2014) 76:176–85. 10.1016/j.biopsych.2013.10.026 24388670

[14] Pizzagalli DA Holmes AJ Dillon DG Goetz EL Birk JL Bogdan R Reduced caudate and nucleus accumbens response to rewards in unmedicated individuals with major depressive disorder. *Am J Psychiatry.* (2009) 166:702–10. 10.1176/appi.ajp.2008.08081201 19411368 PMC2735451

[15] Green IW Pizzagalli DA Admon R Kumar P. Anhedonia modulates the effects of positive mood induction on reward-related brain activation. *Neuroimage.* (2019) 193:115–25. 10.1016/j.neuroimage.2019.02.063 30831312 PMC6813811

[16] Xi C Lai J Du Y Ng CH Jiang J Wu L Abnormal functional connectivity within the reward network: a potential neuroimaging endophenotype of bipolar disorder. *J Affect Disord.* (2021) 280:49–56. 10.1016/j.jad.2020.11.072 33221607

[17] Whittaker JR Foley SF Ackling E Murphy K Caseras X. The functional connectivity between the nucleus accumbens and the ventromedial prefrontal cortex as an endophenotype for bipolar disorder. *Biol Psychiatry.* (2018) 84:803–9. 10.1016/j.biopsych.2018.07.023 30227973 PMC6218647

[18] Russo SJ Nestler EJ. The brain reward circuitry in mood disorders. *Nat Rev Neurosci.* (2013) 14:609–25. 10.1038/nrn3381 23942470 PMC3867253

[19] Sturm V Lenartz D Koulousakis A Treuer H Herholz K Klein JC The nucleus accumbens: a target for deep brain stimulation in obsessive-compulsive– and anxiety-disorders. *J Chem Neuroanat.* (2003) 26:293–9. 10.1016/j.jchemneu.2003.09.003 14729131

[20] Young CB Chen T Nusslock R Keller J Schatzberg AF Menon V. Anhedonia and general distress show dissociable ventromedial prefrontal cortex connectivity in major depressive disorder. *Transl Psychiatry.* (2016) 6:e810. 10.1038/tp.2016.80 27187232 PMC5070048

[21] Liu R Wang Y Chen X Zhang Z Xiao L Zhou Y. Anhedonia correlates with functional connectivity of the nucleus accumbens subregions in patients with major depressive disorder. *Neuroimage Clin.* (2021) 30:102599. 10.1016/j.nicl.2021.102599 33662708 PMC7930634

[22] Gong L He C Zhang H Zhang H Zhang Z Xie C. Disrupted reward and cognitive control networks contribute to anhedonia in depression. *J Psychiatr Res.* (2018) 103:61–8. 10.1016/j.jpsychires.2018.05.010 29783076

[23] Floresco SB. The nucleus accumbens: an interface between cognition, emotion, and action. *Annu Rev Psychol.* (2015) 66:25–52. 10.1146/annurev-psych-010213-115159 25251489

[24] Singh MK Leslie SM Packer MM Weisman EF Gotlib IH. Limbic intrinsic connectivity in depressed and high-risk youth. *J Am Acad Child Adolesc Psychiatry.* (2018) 57:775–785.e3. 10.1016/j.jaac.2018.06.017 30274652 PMC11890206

[25] Bai T Zu M Chen Y Xie W Cai C Wei Q Decreased connection between reward systems and paralimbic cortex in depressive patients. *Front Neurosci.* (2018) 12:462. 10.3389/fnins.2018.00462 30038557 PMC6046444

[26] Furman DJ Hamilton JP Gotlib IH. Frontostriatal functional connectivity in major depressive disorder. *Biol Mood Anxiety Disord.* (2011) 1:11. 10.1186/2045-5380-1-11 22737995 PMC3384258

[27] Rupprechter S Romaniuk L Series P Hirose Y Hawkins E Sandu AL Blunted medial prefrontal cortico-limbic reward-related effective connectivity and depression. *Brain.* (2020) 143:1946–56. 10.1093/brain/awaa106 32385498 PMC7296844

[28] Hutchison RM Womelsdorf T Gati JS Everling S Menon RS. Resting-state networks show dynamic functional connectivity in awake humans and anesthetized macaques. *Hum Brain Mapp.* (2013) 34:2154–77. 10.1002/hbm.22058 22438275 PMC6870538

[29] Li J Duan X Cui Q Chen H Liao W. More than just statics: temporal dynamics of intrinsic brain activity predicts the suicidal ideation in depressed patients. *Psychol Med.* (2019) 49:852–60. 10.1017/S0033291718001502 29909788

[30] Qiao D Zhang A Sun N Yang C Li J Zhao T Altered static and dynamic functional connectivity of habenula associated with suicidal ideation in first-episode, drug-naïve patients with major depressive disorder. *Front Psychiatry.* (2020) 11:608197. 10.3389/fpsyt.2020.608197 33391057 PMC7772142

[31] Shirer WR Ryali S Rykhlevskaia E Menon V Greicius MD. Decoding subject-driven cognitive states with whole-brain connectivity patterns. *Cereb Cortex.* (2012) 22:158–65. 10.1093/cercor/bhr099 21616982 PMC3236795

[32] Marusak HA Calhoun VD Brown S Crespo LM Sala-Hamrick K Gotlib IH Dynamic functional connectivity of neurocognitive networks in children. *Hum Brain Mapp.* (2017) 38:97–108. 10.1002/hbm.23346 27534733 PMC5796541

[33] Faghiri A Stephen JM Wang YP Wilson TW Calhoun VD. Changing brain connectivity dynamics: from early childhood to adulthood. *Hum Brain Mapp.* (2018) 39:1108–17. 10.1002/hbm.23896 29205692 PMC5807176

[34] Damaraju E Allen EA Belger A Ford JM McEwen S Mathalon DH Dynamic functional connectivity analysis reveals transient states of dysconnectivity in schizophrenia. *Neuroimage Clin.* (2014) 5:298–308. 10.1016/j.nicl.2014.07.003 25161896 PMC4141977

[35] Liu F Wang Y Li M Wang W Li R Zhang Z Dynamic functional network connectivity in idiopathic generalized epilepsy with generalized tonic-clonic seizure. *Hum Brain Mapp.* (2017) 38:957–73. 10.1002/hbm.23430 27726245 PMC6866949

[36] Chen H Nomi JS Uddin LQ Duan X Chen H. Intrinsic functional connectivity variance and state-specific under-connectivity in autism. *Hum Brain Mapp.* (2017) 38:5740–55. 10.1002/hbm.23764 28792117 PMC5783325

[37] Li C Xia L Ma J Li S Liang S Ma X Dynamic functional abnormalities in generalized anxiety disorders and their increased network segregation of a hyperarousal brain state modulated by insomnia. *J Affect Disord.* (2019) 246:338–45. 10.1016/j.jad.2018.12.079 30597294

[38] Satterthwaite TD Wolf DH Loughead J Ruparel K Elliott MA Hakonarson H Impact of in-scanner head motion on multiple measures of functional connectivity: relevance for studies of neurodevelopment in youth. *Neuroimage.* (2012) 60:623–32. 10.1016/j.neuroimage.2011.12.063 22233733 PMC3746318

[39] Han S Wang X He Z Sheng W Zou Q Li L Decreased static and increased dynamic global signal topography in major depressive disorder. *Prog Neuropsychopharmacol Biol Psychiatry.* (2019) 94:109665. 10.1016/j.pnpbp.2019.109665 31202912

[40] Zhang Y Mao Z Pan L Ling Z Liu X Zhang J Frequency-specific alterations in cortical rhythms and functional connectivity in trigeminal neuralgia. *Brain Imaging Behav.* (2019) 13:1497–509. 10.1007/s11682-019-00105-8 31209834

[41] Dong C Yang Q Liang J Seger CA Han H Ning Y Impairment in the goal-directed corticostriatal learning system as a biomarker for obsessive-compulsive disorder. *Psychol Med.* (2020) 50:1490–500. 10.1017/S0033291719001429 31272523

[42] Liao W Wu GR Xu Q Ji GJ Zhang Z Zang YF DynamicBC: a MATLAB toolbox for dynamic brain connectome analysis. *Brain Connect.* (2014) 4:780–90. 10.1089/brain.2014.0253 25083734 PMC4268585

[43] Han S Cui Q Wang X Li L Li D He Z Resting state functional network switching rate is differently altered in bipolar disorder and major depressive disorder. *Hum Brain Mapp.* (2020) 41:3295–304. 10.1002/hbm.25017 32400932 PMC7375077

[44] Pujara M Koenigs M. Mechanisms of reward circuit dysfunction in psychiatric illness: prefrontal-striatal interactions. *Neuroscientist.* (2014) 20:82–95. 10.1177/1073858413499407 23924665 PMC4035048

[45] Di Martino A Scheres A Margulies DS Kelly AM Uddin LQ Shehzad Z Functional connectivity of human striatum: a resting state FMRI study. *Cereb Cortex.* (2008) 18:2735–47. 18400794 10.1093/cercor/bhn041

[46] Cauda F Cavanna AE D’Agata F Sacco K Duca S Geminiani GC. Functional connectivity and coactivation of the nucleus accumbens: a combined functional connectivity and structure-based meta-analysis. *J Cogn Neurosci.* (2011) 23:2864–77. 10.1162/jocn.2011.21624 21265603

[47] Diekhof EK Kaps L Falkai P Gruber O. The role of the human ventral striatum and the medial orbitofrontal cortex in the representation of reward magnitude – an activation likelihood estimation meta-analysis of neuroimaging studies of passive reward expectancy and outcome processing. *Neuropsychologia.* (2012) 50:1252–66. 10.1016/j.neuropsychologia.2012.02.007 22366111

[48] Eslinger PJ Damasio AR. Severe disturbance of higher cognition after bilateral frontal lobe ablation: patient EVR. *Neurology.* (1985) 35:1731–41. 10.1212/wnl.35.12.1731 4069365

[49] Camille N Coricelli G Sallet J Pradat-Diehl P Duhamel JR Sirigu A. The involvement of the orbitofrontal cortex in the experience of regret. *Science.* (2004) 304:1167–70. 10.1126/science.1094550 15155951

[50] Pujara MS Wolf RC Baskaya MK Koenigs M. Ventromedial prefrontal cortex damage alters relative risk tolerance for prospective gains and losses. *Neuropsychologia.* (2015) 79:70–5. 10.1016/j.neuropsychologia.2015.10.026 26597003 PMC4679627

[51] Fellows LK Farah MJ. Ventromedial frontal cortex mediates affective shifting in humans: evidence from a reversal learning paradigm. *Brain.* (2003) 126:1830–7. 10.1093/brain/awg180 12821528

[52] Kandilarova S Stoyanov D Stoeva M Latypova A Kherif F. Functional MRI in depression—multivariate analysis of emotional task. *J Med Biol Eng.* (2020) 40:535–44. 10.1007/s40846-020-00547-2

[53] Tremblay L Schultz W. Relative reward preference in primate orbitofrontal cortex. *Nature.* (1999) 398:704–8. 10.1038/19525 10227292

[54] Lopatina N McDannald MA Styer CV Peterson JF Sadacca BF Cheer JF Medial orbitofrontal neurons preferentially signal cues predicting changes in reward during unblocking. *J Neurosci.* (2016) 36:8416–24. 10.1523/JNEUROSCI.1101-16.2016 27511013 PMC4978801

[55] Izquierdo A Suda RK Murray EA. Bilateral orbital prefrontal cortex lesions in rhesus monkeys disrupt choices guided by both reward value and reward contingency. *J Neurosci.* (2004) 24:7540–8. 10.1523/JNEUROSCI.1921-04.2004 15329401 PMC6729636

[56] Cheng W Rolls ET Qiu J Yang D Ruan H Wei D Functional connectivity of the precuneus in unmedicated patients with depression. *Biol Psychiatry Cogn Neurosci Neuroimaging.* (2018) 3:1040–9. 10.1016/j.bpsc.2018.07.008 30243643

[57] Mulinari S. Monoamine theories of depression: historical impact on biomedical research. *J Hist Neurosci.* (2012) 21:366–92. 10.1080/0964704X.2011.623917 22947380

[58] Delva NC Stanwood GD. Dysregulation of brain dopamine systems in major depressive disorder. *Exp Biol Med (Maywood).* (2021) 246:1084–93. 10.1177/1535370221991830 33593109 PMC8113739

[59] Sesack SR Deutch AY Roth RH Bunney BS. Topographical organization of the efferent projections of the medial prefrontal cortex in the rat: an anterograde tract-tracing study with Phaseolus vulgaris leucoagglutinin. *J Comp Neurol.* (1989) 290:213–42. 10.1002/cne.902900205 2592611

[60] Gabbott PL Warner TA Jays PR Salway P Busby SJ. Prefrontal cortex in the rat: projections to subcortical autonomic, motor, and limbic centers. *J Comp Neurol.* (2005) 492:145–77. 10.1002/cne.20738 16196030

[61] Rubio-Casillas A Fernández-Guasti A. The dose makes the poison: from glutamate-mediated neurogenesis to neuronal atrophy and depression. *Rev Neurosci.* (2016) 27:599–622. 10.1515/revneuro-2015-0066 27096778

[62] Sambataro F Wolf ND Pennuto M Vasic N Wolf RC. Revisiting default mode network function in major depression: evidence for disrupted subsystem connectivity. *Psychol Med.* (2014) 44:2041–51. 10.1017/S0033291713002596 24176176

[63] Sharma A Wolf DH Ciric R Kable JW Moore TM Vandekar SN Common dimensional reward deficits across mood and psychotic disorders: a connectome-wide association study. *Am J Psychiatry.* (2017) 174:657–66. 10.1176/appi.ajp.2016.16070774 28135847 PMC5495611

[64] Menon V. Large-scale brain networks and psychopathology: a unifying triple network model. *Trends Cogn Sci.* (2011) 15:483–506. 10.1016/j.tics.2011.08.003 21908230

[65] Aryutova K Paunova R Kandilarova S Stoyanova K Maes MH Stoyanov D. Differential aberrant connectivity of precuneus and anterior insula may underpin the diagnosis of schizophrenia and mood disorders. *World J Psychiatry.* (2021) 11:1274–87. 10.5498/wjp.v11.i12.1274 35070777 PMC8717032

[66] Bartova L Meyer BM Diers K Rabl U Scharinger C Popovic A Reduced default mode network suppression during a working memory task in remitted major depression. *J Psychiatr Res.* (2015) 64:9–18. 10.1016/j.jpsychires.2015.02.025 25801734 PMC4415908

[67] Ho TC Connolly CG Henje Blom E LeWinn KZ Strigo IA Paulus MP Emotion-dependent functional connectivity of the default mode network in adolescent depression. *Biol Psychiatry.* (2015) 78:635–46. 10.1016/j.biopsych.2014.09.002 25483399 PMC4362932

[68] Li B Liu L Friston KJ Shen H Wang L Zeng LL A treatment-resistant default mode subnetwork in major depression. *Biol Psychiatry.* (2013) 74:48–54. 10.1016/j.biopsych.2012.11.007 23273724

[69] Sridharan D Levitin DJ Menon V. A critical role for the right fronto-insular cortex in switching between central-executive and default-mode networks. *Proc Natl Acad Sci USA.* (2008) 105:12569–74. 10.1073/pnas.0800005105 18723676 PMC2527952

[70] Phillips ML Ladouceur CD Drevets WC. A neural model of voluntary and automatic emotion regulation: implications for understanding the pathophysiology and neurodevelopment of bipolar disorder. *Mol Psychiatry.* (2008) 13:829. 10.1038/mp.2008.65 18574483 PMC2745893

[71] Goldin PR McRae K Ramel W Gross JJ. The neural bases of emotion regulation: reappraisal and suppression of negative emotion. *Biol Psychiatry.* (2008) 63:577–86. 10.1016/j.biopsych.2007.05.031 17888411 PMC2483789

[72] McRae K Gross JJ Weber J Robertson ER Sokol-Hessner P Ray RD The development of emotion regulation: an fMRI study of cognitive reappraisal in children, adolescents and young adults. *Soc Cogn Affect Neurosci.* (2012) 7:11–22. 10.1093/scan/nsr093 22228751 PMC3252634

[73] Treadway MT Zald DH. Reconsidering anhedonia in depression: lessons from translational neuroscience. *Neurosci Biobehav Rev.* (2011) 35:537–55. 10.1016/j.neubiorev.2010.06.006 20603146 PMC3005986

[74] Ma Z Li R Yu J He Y Li J. Alterations in regional homogeneity of spontaneous brain activity in late-life subthreshold depression. *PLoS One.* (2013) 8:e53148. 10.1371/journal.pone.0053148 23301035 PMC3534624

[75] Liu F Hu M Wang S Guo W Zhao J Li J Abnormal regional spontaneous neural activity in first-episode, treatment-naive patients with late-life depression: a resting-state fMRI study. *Prog Neuropsychopharmacol Biol Psychiatry.* (2012) 39:326–31. 10.1016/j.pnpbp.2012.07.004 22796277

[76] Smith BJ Monterosso JR Wakslak CJ Bechara A Read SJ. A meta-analytical review of brain activity associated with intertemporal decisions: evidence for an anterior-posterior tangibility axis. *Neurosci Biobehav Rev.* (2018) 86:85–98. 10.1016/j.neubiorev.2018.01.005 29366699

[77] Caseras X Lawrence NS Murphy K Wise RG Phillips ML. Ventral striatum activity in response to reward: differences between bipolar I and II disorders. *Am J Psychiatry.* (2013) 170:533–41. 10.1176/appi.ajp.2012.12020169 23558337 PMC3640293

[78] Singh MK Kelley RG Howe ME Reiss AL Gotlib IH Chang KD. Reward processing in healthy offspring of parents with bipolar disorder. *JAMA Psychiatry.* (2014) 71:1148–56. 10.1001/jamapsychiatry.2014.1031 25142103 PMC11889639

[79] Furl N Averbeck BB. Parietal cortex and insula relate to evidence seeking relevant to reward-related decisions. *J Neurosci.* (2011) 31:17572–82. 10.1523/JNEUROSCI.4236-11.2011 22131418 PMC3474936

[80] Silk JS Siegle GJ Lee KH Nelson EE Stroud LR Dahl RE. Increased neural response to peer rejection associated with adolescent depression and pubertal development. *Soc Cogn Affect Neurosci.* (2014) 9:1798–807. 10.1093/scan/nst175 24273075 PMC4221220

[81] Kumar P Waiter GD Dubois M Milders M Reid I Steele JD. Increased neural response to social rejection in major depression. *Depress Anxiety.* (2017) 34:1049–56. 10.1002/da.22665 28632961

[82] Jankowski KF Batres J Scott H Smyda G Pfeifer JH Quevedo K. Feeling left out: depressed adolescents may atypically recruit emotional salience and regulation networks during social exclusion. *Soc Cogn Affect Neurosci.* (2018) 13:863–76. 10.1093/scan/nsy055 30059994 PMC6123522

[83] Spreckelmeyer KN Krach S Kohls G Rademacher L Irmak A Konrad K Anticipation of monetary and social reward differently activates mesolimbic brain structures in men and women. *Soc Cogn Affect Neurosci.* (2009) 4:158–65. 10.1093/scan/nsn051 19174537 PMC2686229

[84] Wilson RP Colizzi M Bossong MG Allen P Kempton M Bhattacharyya S. The neural substrate of reward anticipation in health: a meta-analysis of fMRI findings in the monetary incentive delay task. *Neuropsychol Rev.* (2018) 28:496–506. 10.1007/s11065-018-9385-5 30255220 PMC6327084

[85] Conio B Martino M Magioncalda P Escelsior A Inglese M Amore M Opposite effects of dopamine and serotonin on resting-state networks: review and implications for psychiatric disorders. *Mol Psychiatry.* (2020) 25:82–93. 10.1038/s41380-019-0406-4 30953003

[86] Soehner AM Bertocci MA Manelis A Bebko G Ladouceur CD Graur S Preliminary investigation of the relationships between sleep duration, reward circuitry function, and mood dysregulation in youth offspring of parents with bipolar disorder. *J Affect Disord.* (2016) 205:144–53. 10.1016/j.jad.2016.03.074 27442458 PMC5129838

[87] Campos M Breznen B Bernheim K Andersen RA. Supplementary motor area encodes reward expectancy in eye-movement tasks. *J Neurophysiol.* (2005) 94:1325–35. 10.1152/jn.00022.2005 15843484

[88] Yang XH Tian K Wang DF Wang Y Cheung EFC Xie GR Anhedonia correlates with abnormal functional connectivity of the superior temporal gyrus and the caudate nucleus in patients with first-episode drug-naive major depressive disorder. *J Affect Disord.* (2017) 218:284–90. 10.1016/j.jad.2017.04.053 28478357

[89] Kaiser RH Whitfield-Gabrieli S Dillon DG Goer F Beltzer M Minkel J Dynamic resting-state functional connectivity in major depression. *Neuropsychopharmacology.* (2016) 41:1822–30. 10.1038/npp.2015.352 26632990 PMC4869051

[90] Pang Y Chen H Wang Y Long Z He Z Zhang H Transdiagnostic and diagnosis-specific dynamic functional connectivity anchored in the right anterior insula in major depressive disorder and bipolar depression. *Prog Neuropsychopharmacol Biol Psychiatry.* (2018) 85:7–15. 10.1016/j.pnpbp.2018.03.020 29608925

[91] Pang Y Zhang H Cui Q Yang Q Lu F Chen H Combined static and dynamic functional connectivity signatures differentiating bipolar depression from major depressive disorder. *Aust N Z J Psychiatry.* (2020) 54:832–42. 10.1177/0004867420924089 32456443

[92] Rashid B Arbabshirani MR Damaraju E Cetin MS Miller R Pearlson GD Classification of schizophrenia and bipolar patients using static and dynamic resting-state fMRI brain connectivity. *Neuroimage.* (2016) 134:645–57. 10.1016/j.neuroimage.2016.04.051 27118088 PMC4912868

[93] Yan CG Chen X Li L Castellanos FX Bai TJ Bo QJ Reduced default mode network functional connectivity in patients with recurrent major depressive disorder. *Proc Natl Acad Sci USA.* (2019) 116:9078–83. 10.1073/pnas.1900390116 30979801 PMC6500168

[94] Nifosì F Toffanin T Follador H Zonta F Padovan G Pigato G Reduced right posterior hippocampal volume in women with recurrent familial pure depressive disorder. *Psychiatry Res.* (2010) 184:23–8. 10.1016/j.pscychresns.2010.05.012 20817488

